# Early assessment of *KRAS* mutation in cfDNA correlates with risk of progression and death in advanced non-small-cell lung cancer

**DOI:** 10.1038/s41416-020-0833-7

**Published:** 2020-05-07

**Authors:** Elisabetta Zulato, Ilaria Attili, Alberto Pavan, Giorgia Nardo, Paola Del Bianco, Andrea Boscolo Bragadin, Martina Verza, Lorenza Pasqualini, Giulia Pasello, Matteo Fassan, Fiorella Calabrese, Valentina Guarneri, Alberto Amadori, PierFranco Conte, Stefano Indraccolo, Laura Bonanno

**Affiliations:** 1 Immunology and Molecular Oncology Unit, Istituto Oncologico Veneto IOV – IRCCS, Padova, Italy; 2 Medical Oncology 2, Istituto Oncologico Veneto IOV – IRCCS, Padova, Italy; 3 Department of Surgery, Oncology and Gastroenterology, Università degli Studi di Padova, Padova, Italy; 4 Clinical Research Unit, Istituto Oncologico Veneto IOV – IRCCS, Padova, Italy; 5 Surgical Pathology Unit, Department of Medicine (DIMED), Università degli Studi di Padova, Padova, Italy; 6 Pathology Unit, Department of Cardiothoracic Sciences, Università degli Studi di Padova, Padova, Italy

**Keywords:** Non-small-cell lung cancer, Tumour biomarkers

## Abstract

**Background:**

Liquid biopsy has the potential to monitor biological effects of treatment. *KRAS* represents the most commonly mutated oncogene in Caucasian non-small-cell lung cancer (NSCLC). The aim of this study was to explore association of dynamic plasma *KRAS* genotyping with outcome in advanced NSCLC patients.

**Methods:**

Advanced NSCLC patients were prospectively enrolled. Plasma samples were collected at baseline (T1), after 3 or 4 weeks, according to treatment schedule (T2) and at first radiological restaging (T3). Patients carrying *KRAS* mutation in tissue were analysed in plasma with droplet digital PCR. Semi-quantitative index of fractional abundance of mutated allele (MAFA) was used.

**Results:**

*KRAS*-mutated cohort included 58 patients, and overall 73 treatments (*N* = 39 chemotherapy and *N* = 34 immune checkpoint inhibitors) were followed with longitudinal liquid biopsy. Sensitivity of *KRAS* detection in plasma at baseline was 48.3% (95% confidence interval (CI): 35.0–61.8). *KRAS* mutation at T2 was associated with increased probability of experiencing progressive disease as best radiological response (adjusted odds ratio: 7.3; 95% CI: 2.1–25.0, *p* = 0.0016). Increased MAFA (T1–T2) predicted shorter progression-free survival (adjusted hazard ratio (HR): 2.1; 95% CI: 1.2–3.8, *p* = 0.0142) and overall survival (adjusted HR: 3.2; 95% CI: 1.2–8.4, *p* = 0.0168).

**Conclusions:**

Longitudinal analysis of plasma *KRAS* mutations correlated with outcome: its early assessment during treatment has great potentialities for monitoring treatment outcome in NSCLC patients.

## Background

The outcome of advanced non-small-cell lung cancer (NSCLC) has been substantially improving in the latest years, in parallel with increased knowledge of its molecular bases and the introduction of immune checkpoint inhibitors (ICIs) in clinical practice.^1^ Given the availability of new therapeutic options, the possibility of monitoring disease burden and effects of treatment by liquid biopsy is acquiring more and more relevance.

Detection of cancer mutations in circulating cell-free DNA (cfDNA) has been investigated by various techniques in several solid tumours and it is meanwhile a diagnostic tool in advanced NSCLC for two indications: detection of *EGFR* sensitising mutations at baseline, when tissue analysis is not possible, and detection of acquired resistance *EGFR* mutations at progression on EGFR inhibitors.^2^ Further potential applications including multiple cfDNA genetic testing have already been included in NCCN (National Comprehensive Cancer Network) guidelines, even though tissue genotyping remains the gold standard for diagnosis.^3^ The feasibility to detect and characterise cancer mutations in cfDNA has the potential to shed light on tumour heterogeneity, acquired resistance mechanisms and provide dynamic information on biological effects of anti-cancer treatment.^4,5^ Theoretically, their detection in plasma could be relatively less influenced by circulating non-tumour DNA and might be more specific than other circulating biomarkers,^6,7^ even though the presence of genetic alterations in peripheral blood cells stemming from clonal haematopoiesis could represent a potential challenge.^6,7^

Specifically, in the field of immunotherapy, analysis of tumour-specific genetic alterations in cfDNA may help to discriminate pseudo-progression from true progression during treatment with ICIs^8^ and the dynamic quantification of tumour-specific genetic alterations may provide more complete information, acting as potential predictive biomarker.

We performed a prospective screening of genetic alterations in tumour tissue of patients with *EGFR-ALK-ROS1* wild-type advanced NSCLC. Here we describe the cohort of *KRAS*-mutated patients treated with chemotherapy or ICIs. Mutations in *KRAS* oncogene are the most prevalent genetic alterations in Caucasian NSCLC. While association with prognosis is controversial,^9^ effective KRAS-targeted therapies were not available until recently, when evidence has emerged about therapeutic activity of the specific inhibitor AMG-510 in G12C *KRAS*-mutated NSCLC.^10^

We monitored the presence and the quantitative variation of *KRAS* mutations in plasma samples collected at pre-planned time-points during treatment using droplet digital PCR (ddPCR). Tumour-specific genetic alterations analysed in plasma were used as a surrogate marker of tumour load with the aim to monitor biological effects of treatment and explore the impact of their variation on outcome.

## Methods

### Patients

We prospectively enrolled advanced NSCLC patients starting systemic treatment at our Institution between January 2017 and August 2019. Eligibility criteria were availability of tumour biopsy material collected before starting any treatment, the planning of systemic treatment and the possibility of adequate clinical and radiological follow-up.

Tissue molecular analyses were performed at baseline according to standard clinical practice and patients carrying *EGFR* sensitising mutations or *ALK, ROS1* rearrangements were excluded from the analysis.

Patients were treated according to clinical practice with chemotherapy or ICIs, and palliative local treatment was allowed according to treating physician’s choice. During systemic treatment, radiological evaluation was performed with iodine contrast computed tomography scan at baseline and during treatment according to clinical practice.

The ethics committee of Istituto Oncologico Veneto evaluated and approved study design and informed consent (2016/82, 12 December 2016). Written informed consent was obtained from all patients before study entry. The study was conducted in accordance with the precepts of the Declaration of Helsinki .

### Tissue genetic analysis

Clinical diagnostic tissue genotyping was performed using the Sequenom MassARRAY^®^ (Sequenom MA) Myriapod Lung Status Kit (Diatech Pharmacogenetics SRL, Jesi, Italy) (Supplementary Table 1A).

In the absence of any previously determined mutations among those screened according to clinical practice, tissue genetic alterations were screened by next-generation sequencing (NGS)^7^ (Illumina, San Diego, CA, USA) using a custom panel of 30 lung cancer related-genes that covers 25,741 bp for a total of 284 amplicons (Supplementary Table 1B).

All formalin-fixed, paraffin-embedded (FFPE) samples were evaluated by a pathologist in order to assess the tumour tissue quality and quantity.

Four FFPE sections were used for genomic DNA (gDNA) extraction, using the Qiamp DNA Micro Kit (Qiagen, Hilden, Germany). gDNA was quantified with Qubit 3.0 Fluorometer (Invitrogen, Carlsbad, CA), and stored at −20 °C before use. According to the DNA quality assessed through the Trusses FFPE DNA Library Prep QC Kit (Illumina), sequencing libraries were generated from 80 to 200 ng DNA adopting the TruSeq Custom Amplicon Low Input Kit with a Dual Strand Design (Illumina). Ten samples per run were sequenced using the MiSeq Reagent Kit v3 on the Illumina MiSeq Sequencer in paired-end mode (2 × 125 cycles). FASTQ files were processed using the SOPHiA DDM platform (SOPHiA GENETICS SA, Saint Sulpice, Switzerland). Variants selection was performed considering a depth value ≥400 reads and a variant allele fraction ≥1%. Only the canonical variants were taken into account as possible trackable mutations.

### Plasma sample collection and DNA extraction

Plasma samples were collected at the time of first administration of systemic treatment (baseline, T1), after 3 or 4 weeks of treatment (according to the treatment schedule) (3 ± 1 weeks, T2), and at first radiological restaging (T3) (Supplementary Fig. 1a). Samples were collected on the same day of administration of first cycle of treatment (T1), second cycle of treatment (T2) or during the clinical visit following radiological re-evaluation (T3). When patients enrolled in the study started a new systemic treatment after progressive disease (PD), they were re-considered for longitudinal analysis, and a new baseline plasma sample (T1) was collected.

At each time-point, 20 ml of blood was collected in two Helix cfDNA Stabilization tubes (Diatech Pharmacogenetics SRL) and processed within 24–72 h. Blood was centrifuged at 2000 × *g* for 10 min at 4 °C. Next, the supernatant was centrifuged at 20,000 × *g* for 10 min and plasma was stored at −80 °C. cfDNA was extracted from a total of 1 ml of plasma using the Maxwell^®^ RSC ccfDNA Plasma Kit (Promega, Madison, Wisconsin, USA), eluted into 60 μl of buffer and stored at −20 °C.

### Analysis of *KRAS* mutations in plasma

ddPCR was carried out on the QX200 ddPCR system (Bio-Rad Laboratories).^11^ ddPCR probes matching *KRAS* codon G12/G13 and Q61 mutations were purchased from Bio-Rad (the ddPCR *KRAS* G12/G13 Screening Kit #1863506 and the ddPCR™ *KRAS* Q61 Screening Kit #12001626). These ddPCR assays detect mutations in *KRAS* codon G12/G13 (G12A, G12C, G12D, G12R, G12S, G12V, G13D) and in codon Q61 (Q61H, Q61K, Q61L, Q61R), but they are not able to distinguish among individual mutations. *KRAS* mutations matching this list and identified in tumour tissue by Sequenom MassARRAY^®^ or NGS were tracked in plasma by ddPCR.

Twenty microlitres (μl) of PCR reaction was prepared according to the manufacturer’s instructions with 7.5 μl cfDNA. Each sample was analysed in triplicate. PCR reaction was portioned into a mean of 15,000 droplets per sample using QX100 Droplet Generation (Bio-Rad), transferred to the Bio-Rad QX200 droplet reader and fluorescence intensity was analysed by QuantaSoft 1.7.4.0917 software (Bio-Rad). In each test, at least three control wells with a negative *KRAS* cfDNA, one negative control well without DNA and one positive control were included. Amplification of each sample was calculated as the number of total droplets (wild-type + mutated droplets) and a mean of 1480 droplets per sample was observed. A cut-off of three droplets is used to call a sample mutant, according to the Poisson’s law of small numbers (as reported in the manufacturer’s instructions). Semi-quantitative index of fractional abundance of mutated allele (MAFA) was calculated as follows: [no. of mutated droplets/(wild-type + mutated droplets)].^11^ ddPCR *KRAS* Screening Multiplex Kit allows detection down to a MAFA of 0.2% for multiple mutations in cfDNA samples, as reported in the manufacturer’s instructions.

Representative cases positive for *KRAS* mutation at T2 or T3, but negative at previous baseline (T1) evaluation, were also analysed in peripheral blood cells by ddPCR in order to exclude potential confounding impact of clonal haematopoiesis.^7,12^ All the samples analysed were found negative for the presence of *KRAS* mutation (data not shown).

### Statistical analyses

To assess the sensitivity of ddPCR in detecting *KRAS* mutations in plasma in advanced NSCLC patients, the sample size was calculated considering that 41 mutated patients allow to estimate a 95% confidence interval (95% CI) of length 30% for a sensitivity of detecting *KRAS* mutation at baseline of 60%. Because of the exploratory nature of dynamic analysis of liquid biopsy, no sample size calculation was defined before study initiation.

Results of liquid biopsy were considered both as a static parameter (presence *versus* absence of *KRAS* mutation at each time-points) and as a dynamic parameter (increase of MAFA from baseline *versus* stable/decreasing value) and correlated with clinical outcomes.^11,13^ MAFA variations were considered as difference from baseline to different time-points (T1–T2 and T1–T3) and any change was considered for statistical correlations.

Quantitative variables were summarised as median and interquartile range (IQ), categorical variables as counts and percentages. The association with clinical variables was verified using *χ*^2^ or Fisher’s exact test as appropriate. The median follow-up time was based on the reverse Kaplan–Meier estimator.

Radiological response (RR) was assessed by using RECIST criteria v1.1. Progression-free survival (PFS) was calculated as the time from the beginning of the systemic treatment (corresponding to T1—the time of the baseline sample draw) to radiological PD or death for any cause. Overall survival (OS) was calculated as the time from the beginning of the systemic treatment to death from any cause. Patients who did not develop an event during the study period were censored at the date of last observation. Median PFS and OS were estimated using the Kaplan–Meier method.

Patients were evaluated for study endpoints every time they started a new systemic treatment.

The impact of clinical characteristics, *KRAS* mutation and change from baseline in *KRAS* MAFA on the probability of radiological PD was evaluated in univariate mixed-effects logistic regression models to account the multilevel structure of the data. Separate multiple mixed-effects logistic regression models were estimated for *KRAS* mutation at each time-point, and change in *KRAS* MAFA from baseline together with clinical factors resulted significant at univariate analysis.

The association of clinical and biological variables with PFS and OS was investigated through univariate mixed-effects Cox proportional hazards regression models to handle the clustered nature of the data and the time-varying covariates, after checking any deviation from the proportional hazards assumption.^14^ A robust variance ‘sandwich' estimator was used to adjust for the within-subject correlation. Hazard ratios (HRs) for *KRAS* mutation at each time-point and variation of *KRAS* MAFA from baseline were also adjusted for any clinical factor resulting statistically significant at univariate analysis.

To assess potential predictive value of the presence of *KRAS* mutation at T1, T2, T3 and MAFA modification during treatment, interaction test with different treatment received (chemotherapy *versus* ICIs) was used.

All statistical tests used a two-sided 5% significance level and association measures were provided with their 95% CI. Statistical analyses were performed using the SAS statistical package (SAS, rel. 9.4; SAS Institute Inc.) and RStudio (RStudio: Integrated Development for R. RStudio, Inc., Boston, MA).

## Results

### Patients and tissue genotyping

Study design is summarised in Supplementary Fig. 1a.

A total of 254 patients with *EGFR-ALK-ROS1* wild-type advanced NSCLC were enrolled and, until now, 138 patients completed tissue genetic screening (Supplementary Table 2). Sixty patients carried *KRAS* mutation in tissue: 49 patients in codon 12, five in codon 13, while codon 61 was altered in six cases (Supplementary Fig. 1b; Supplementary Table 2). Fifty-eight *KRAS*-mutated patients were considered for the analysis. Patient #9 and #107 were not considered for the analysis, because their mutations (*KRAS* G12F and G13S, respectively) were not included the ddPCR Kit used for the study (Supplementary Table 2).

Clinical features and treatment details are summarised in Table 1. When patients enrolled in the study started a new systemic treatment after PD, they were re-considered for longitudinal analysis. Overall, *KRAS*-mutated patients received 73 treatments during the period of observation of the study: nine patients changed treatment once and three patients twice. Thirty-nine chemotherapy and 34 ICIs treatments were followed (Table 1b).

**Table 1:** Clinical features of *KRAS*-mutated patients.

(a) Clinical characteristics of *KRAS*-mutated patients	
Age at diagnosis (years)	
Median (Q1–Q3)	67.5 (61–73)
Follow-up (months)	
Median (Q1–Q3)	13.1 (10.1–15.4)
Gender	
Male	31 (53.4%)
Female	27 (46.6%)
Smoking	
No	8 (13.8%)
Yes	21 (36.2%)
former	29 (50.0%)
PS	
0	26 (44.8%)
1	28 (48.3%)
2	4 (6.9%)
Total	58

(b) Disease burden and treatment characteristics across lines	
Extra-thoracic sites	
No	31 (42.5%)
1	24 (32.9%)
>1	18 (24.7%)
Number of metastatic sites	
0–1	38 (52.1%)
2–4	35 (47.9%)
Bone/liver	
No	47 (64.4%)
Bone	9 (12.3%)
Liver	9 (12.3%)
Both	8 (11.0%)
Treatment lines	
1	43 (58.9%)
>1	30 (41.1%)
Type of treatment	
Immunotherapy	34 (46.6%)
Nivolumab	12 (16.4%)
Pembrolizumab	18 (24.7%)
Atezolizumab	4 (5.5%)
Mono-chemotherapy	14 (19.2%)
Docetaxel-Nintedanib	2 (2.7%)
Docetaxel	5 (6.9%)
Gemcitabine	2 (2.7%)
Vinorelbine	5 (6.9%)
Platinum-doublet	25 (34.2%)
Platinum-pemetrexed	17 (23.3%)
platinum-gemcitabine	6 (8.2%)
carboplatinum-paclitaxel	2 (2.7%)
Total	73

The impact of clinical features on RR and outcome is depicted in Supplementary Tables 3–5.

The median follow-up of our study population was 13.1 (95% CI: 10.1–15.4) months.

### Baseline liquid biopsy

Fifty-eight *KRAS*-mutated patients were tested in plasma on the same day of first administration of systemic treatment (baseline, T1). *KRAS* mutation was found in 28 cases (Supplementary Fig. 1b; Supplementary Table 6). The sensitivity of *KRAS* mutation detection in plasma at baseline was 48.3% (95% CI: 35.0–61.8).

In our series no significant association was found between the presence and relative quantification of *KRAS* mutation in plasma and tumour burden, in terms of the number of metastatic sites or the presence of bone and/or liver metastases (Supplementary Table 7).

The detection of mutation in plasma at baseline was also not confirmed as negative prognostic marker. The presence of the mutation in plasma was not statistically associated with RR and PFS. The effect on OS was not confirmed when considering potential confounding effects of confirmed clinical prognostic markers (Table 2 and Supplementary Table 5).

**Table 2:** Association of liquid biopsy results and radiological response, progression free survival and overall survival.

(a) Association of *KRAS* mutation status and RR							
	PD/*N*	OR (PD)	95% CI	*p* Value	Adjusted OR^a^	95% CI	*p* Value
T1							
* KRAS* positive	16/35	2.0	0.8–5.3	0.1534	1.8	0.6–5.5	0.3062
* KRAS* negative	11/37	1			1		
T2							
* KRAS* positive	15/28	5.3	2.0–13.9	0.0008	7.3	2.1–25.0	**0.0016**
* KRAS* negative	7/38	1			1		
T3							
* KRAS* positive	12/21	7.8	2.1–29.2	0.0023	9.8	2.4–40.3	**0.0015**
* KRAS* negative	4/40	1			1		
Δ (T1–T2)							
MAFA increase	9/17	3.5	1.1–10.9	0.0279	8.0	1.5–42.8	**0.0145**
MAFA stable/decrease	13/49	1			1		
Δ (T1–T3)							
MAFA increase	10/16	10.8	2.5–47.2	0.0015	14.2	3.0–68.7	**0.0009**
MAFA stable/decrease	6/45	1			1		

(b) Association of *KRAS* mutation status and PFS								
	PD/*N*	Median PFS (95% CI)	HR	95% CI	*p* Value	Adjusted HR^b^	95% CI	*p* Value
T1								
* KRAS* positive	28/35	5.6 (3.0–10.4)	1.0	0.6–1.7	0.9483	0.9	0.5–1.6	0.7302
* KRAS* negative	33/38	7.2 (5.4–9.2)	1			1		
T2								
* KRAS* positive	24/28	4.7 (2.8–8.6)	1.4	0.8–2.3	0.2483	1.4	0.8–2.4	0.1891
* KRAS* negative	31/39	8.1 (5.8–10.3)	1			1		
T3								
* KRAS* positive	20/21	3.8 (2.1–6.7)	2.9	1.5–5.9	0.0025	2.3	1.1–4.6	**0.0198**
* KRAS* negative	30/40	9.9 (7.9–13.1)	1			1		
Δ (T1–T2)								
MAFA increase	16/17	5.2 (2.1–9.2)	1.6	0.9–2.9	0.0925	2.1	1.2–3.8	**0.0142**
MAFA stable/decrease	39/50	8.0 (5.6–9.9)	1			1		
Δ (T1–T3)								
MAFA increase	15/16	2.8 (1.8–5.7)	3.3	1.4–7.5	0.0055	2.8	1.3–6.3	**0.0119**
MAFA stable/decrease	35/45	9.9 (7.9–11.0)	1			1		

(c) Association of *KRAS* mutation status and OS								
	Death/*N*	Median OS (95% CI)	HR	95% CI	*p* Value	Adjusted HR^c^	95% CI	*p* Value
T1								
* KRAS* positive	19/35	11.2 (4.8–22.1)	2.4	1.1–5.1	0.0231	2.1	0.9–4.7	0.0746
* KRAS* negative	11/38	–	1					
T2								
* KRAS* positive	16/28	10.8 (4.5–22.1)	3.0	1.4–6.6	0.0066	3.1	1.4–7.0	**0.0063**
* KRAS* negative	11/41	–	1					
T3								
* KRAS* positive	13/21	10.7 (5.6–12.9)	6.6	2.4–18.2	0.0002	5.0	1.6–15.0	**0.0044**
* KRAS* negative	6/40	–	1					
Δ (T1–T2)								
MAFA increase	10/17	10.7 (3.4–)	2.4	1.1–5.4	0.0313	3.2	1.2–8.4	**0.0168**
MAFA stable/decrease	15/50	22.1 (11.9-)	1					
Δ (T1–T3)								
MAFA increase	10/16	8.3 (4.4–11.2)	4.5	1.7–11.8	0.0027	3.9	1.5–10.6	**0.0067**
MAFA stable/decrease	9/45	22.1 (21.5-)	1					

*PFS* progression-free survival, *OS* overall survival, *RR* radiological response, *MAFA* mutated allele fractional abundance, *PD* progressive disease, *N* number of treatments, *AUC* area under the curve, *CI* confidence interval, *OR* odds ratio, HR hazard ratio.

Bold values indicate statistical significance *p* < 0.05.

^a^OR adjusted for clinical prognostic factors (see Supplementary Table 3).

^b^HR adjusted for clinical significant factors (see Supplementary Table 4).

^c^HR adjusted for clinical significant factors (see Supplementary Table 5).

### Monitoring of plasma genotyping and RR

We evaluated the correlation of the results of liquid biopsy with RR both considering liquid biopsy as a static parameter (presence *versus* absence of *KRAS* mutation) and as a dynamic parameter (increase of MAFA *versus* stable/decreasing value).

Even adjusted for clinical prognostic factors, the presence of *KRAS* mutation detected at T2 (Supplementary Fig. 2) and at T3 was statistically associated with a higher risk of experiencing PD as best RR: OR = 7.3 (95% CI: 2.1–25.0, *p* = 0.0016) and OR = 9.8 (95% CI: 2.4–40.3, *p* = 0.0015), respectively (Table 2a).

Interestingly, any increase in MAFA from T1 to T2 and from T1 to T3 was consistently associated with risk of PD: adjusted OR was 8 (95% CI: 1.5–42.8, *p* = 0.0145) for MAFA variations from T1 to T2, and 14.2 (95% CI: 3.0–68.7, *p* = 0.0009) for MAFA variations from T1 to T3 (Table 2b).

### Monitoring of plasma genotyping and PFS

**Fig. 1:**
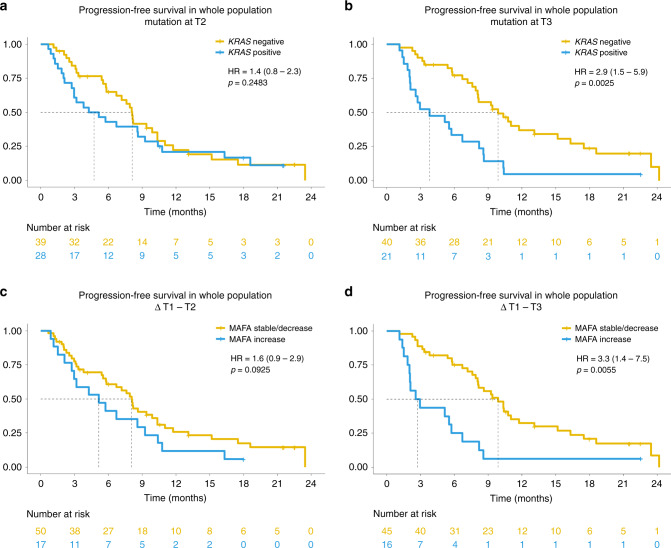
Association of liquid biopsy results and progression-free survival (PFS). Kaplan–Meier curves showing progression-free survival (PFS) of the overall study population according to the presence or the variation in mutated allele fractional abundance (MAFA) of *KRAS* mutation. The hazard ratios (HR) with 95% confidence interval (CI) and *p* values are also reported in the figure. **a** PFS according to the presence of *KRAS* mutation at T2; **b** PFS according to the presence of *KRAS* mutation at T3; **c** PFS according to MAFA variation from baseline (T1) to T2; **d** PFS according to MAFA variation from T1 to T3.

After observing the effect of liquid biopsy performed at baseline, we tested the impact of its presence and variation at early time-points during treatment (Table 2b and Fig. 1).

The presence of *KRAS* mutation in plasma at T3 was associated with shorter PFS with HR adjusted for clinical prognostic factors of 2.3 (95% CI: 1.1–4.6, *p* = 0.0198) (Table 2b and Fig. 1b; Supplementary Table 3). Patients with *KRAS* mutation in plasma at T3 had a median PFS of 3.8 (95% CI: 2.1–6.7) *versus* 9.9 (7.9–13.1) months for patients with negative liquid biopsy (Table 2b and Fig. 1b).

Dynamic variations from T1 to T2 and from T1 to T3 had significant impact on PFS of the study population with statistical significance: adjusted HR was 2.1 (95% CI: 1.2–3.8, *p* = 0.0142) for MAFA increase from T1 to T2 and 2.8 (95% CI: 1.3–6.3, *p* = 0.0119) for increase from T1 to T3 (Table 2b and Fig. 1c–d; Supplementary Table 4).

Median PFS for patients experiencing increase in MAFA (T1–T2) was 5.2 (95% CI: 2.1–9.2) months *versus* 8 (95% CI: 5.6–9.9) for patients with decreased/stable MAFA (T1–T2) (Table 2 and Fig. 1).

### Monitoring of plasma genotyping and OS

**Fig. 2:**
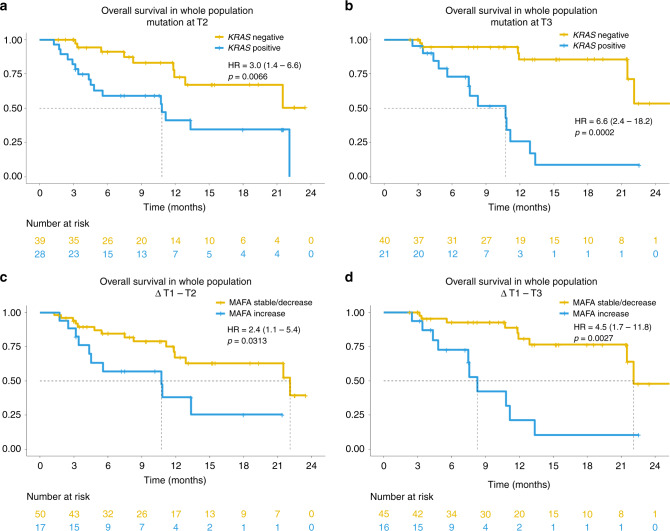
Association of liquid biopsy results and overall survival (OS). Kaplan–Meier curves showing overall survival (OS) of the overall study population according to the presence or the variation in mutated allele fractional abundance (MAFA) of *KRAS* mutation. The hazard ratios with 95% confidence interval and *p* values are also reported in figure. **a** OS according to the presence of *KRAS* mutation at T2; **b** OS according to the presence of *KRAS* mutation at T3; **c** OS according to MAFA variation from baseline (T1) to T2; **d** OS according to MAFA variation from T1 to T3.

The presence of *KRAS* mutation in plasma at early time-points during treatment and its increase from baseline was associated with worse OS, even when considering potential confounding effects of clinical prognostic factors (Table 2 and Fig. 2; Supplementary Table 5).

Detection of *KRAS* mutation in plasma at T2 was associated with shorter OS with adjusted HR of 3.1 (95% CI: 1.4–7.0, *p* = 0.0063) (Table 2c; Fig. 2a). Consistently, the adjusted HR for the positivity at T3 was 5.0 (95% CI: 1.6–15.0, *p* = 0.0044) (Table 2c; Fig. 2b).

When we considered dynamic analysis of the *KRAS* mutation in plasma, we observed that patients experiencing MAFA increase T1–T2 and T1–T3 had increased risk of death, with adjusted HR of 3.2 (95% CI: 1.2–8.4, *p* = 0.0168) and 3.9 (95% CI: 1.5–10.6, *p* = 0.0067), respectively (Table 2c; Fig. 2c, d).

### Liquid biopsy and clinical outcome according to treatment

Patients enrolled in this study were treated in different lines of treatment (Table 1B). In any case, statistical analyses demonstrated that the impact of static and dynamic analysis on outcome was independent from line of treatment (first line *versus* subsequent line of treatment) (data not shown). Patients included in the analysis received different kinds of treatment: we followed longitudinally patients receiving chemotherapy (*N* = 39) and ICIs (*N* = 34). Post hoc analyses depicted in Supplementary Fig. 3 and interaction test analyses demonstrated that the impact of static and dynamic analysis during treatment on outcome was independent from type of treatment (chemotherapy *versus* ICIs) (Supplementary Figs. 3–5).

**Fig. 3:**
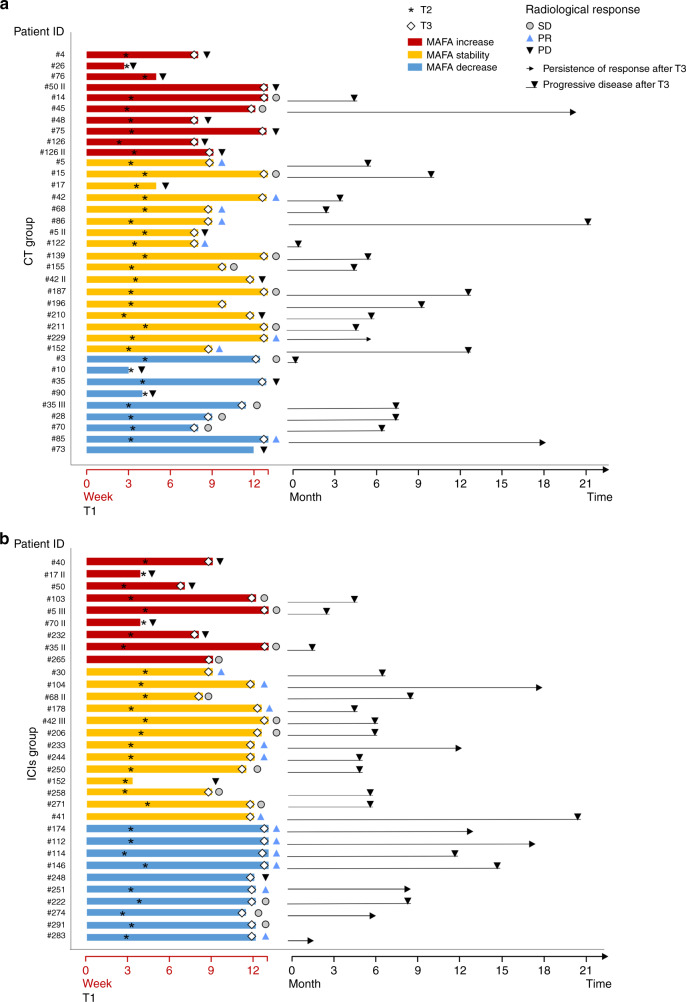
Clinical benefit according to longitudinal liquid biopsy analysis in patients treated with chemotherapy (CT) or immune checkpoint inhibitors (ICIs). The figure depicts the duration of treatment, persistence of clinical benefit and radiological response according to MAFA variation from T1 to T3 in patients treated with CT (**a**) or ICIs (**b**). MAFA variation from T1 to T3 are plotted as horizontal bars; the timing of T2 and radiological evaluation (corresponding to T3) are indicated by the asterisk and diamond, respectively. Radiological response at T3 is reported, as specified in the figure caption. Lines interrupted by black triangle and arrows represent progressive disease and persistence of response after T3, respectively. PD: progressive disease; SD: stable disease; PR: partial response.

Details about RR and duration of response and results of liquid biopsy according to treatment are depicted in Fig. 3 and Supplementary Fig. 2.

Among patients treated with chemotherapy, the presence of *KRAS* mutation at T2 and its increase from T1 to T2 were associated with an increased risk of death with an HR of 4.5 (95% CI: 1.5–13.9) and 8.3 (95% CI: 2.1–32.3), respectively (Supplementary Figs. 3,  4e and 6g).

When we considered patients treated with ICIs, the evaluation of liquid biopsy at T3 significantly impacts on PFS: HR of 5.5 (95% CI: 2.5–11.9) for the presence of *KRAS* mutation at T3 and 5.4 (95% CI: 2.5–11.9) for MAFA increase T1–T3 (Supplementary Figs. 3 and 5b, d). A clear trend for shorter OS was also seen when we considered the increase T1–T2 (Supplementary Figs. 3 and 5g) and became statistically significant when we evaluated the impact of the presence of *KRAS* mutation at T3 and its increase T1–T3 (*p* = 0.0031 and *p* = 0.018; Supplementary Figs. 3 and 5f, h).

**Fig. 4:**
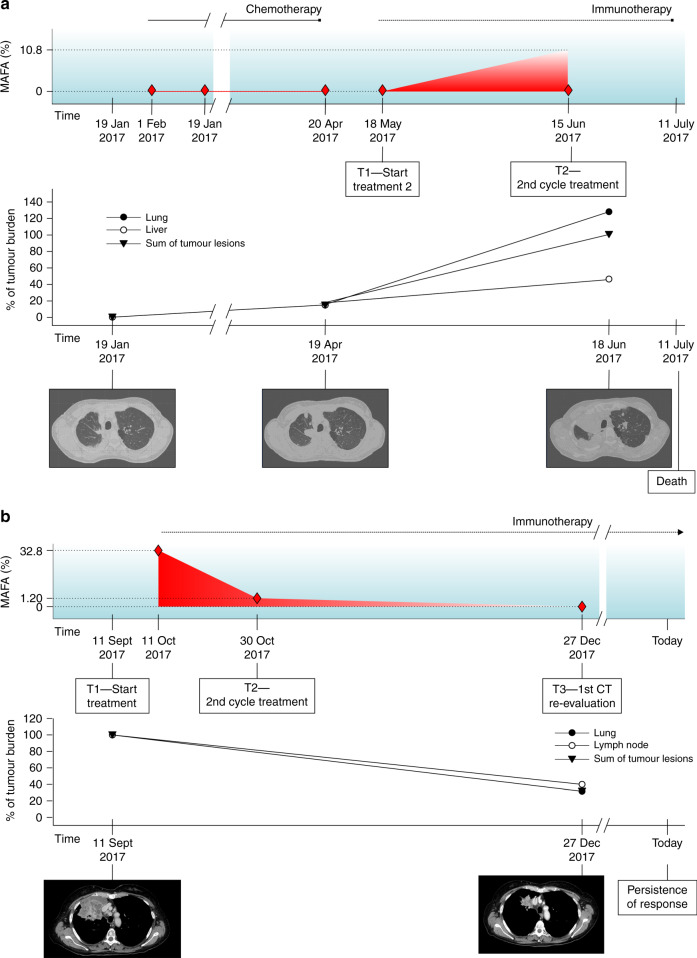
Specific pattern of response according to longitudinal liquid biopsy analysis in patients treated with immune checkpoint inhibitors (ICIs). **a** The figure shows the dynamic monitoring of patient #17 II harbouring *KRAS* G12D mutation, during immunotherapy. She was a 48-year-old woman, heavy smoker. Baseline CT scan was acquired 28 days before the administration of nivolumab and first liquid biopsy (T1). After 4 weeks (T2), liquid biopsy revealed an increase in allelic fraction (10.8% *versus* 0%). The patient thus presented worsening clinical conditions and a CT scan was performed ahead of schedule showing disease progression. Patient died only 23 days later. Radiological review of CT scans confirmed that the radiological changes are consistent with the definition of ’hyper-progression’. Diamond symbols indicate liquid biopsies. **b** The figure shows dynamic monitoring of patient #112, a woman with active smoking habit. At baseline, liquid biopsy showed a MAFA of 32.8%. After 3 weeks (T2), MAFA dropped to 1.2% and liquid biopsy was negative for *KRAS* mutation at the time of radiological evaluation (T3). The patient had partial response (T3) and she is currently under treatment, experiencing persistent clinical benefit. Diamond symbols indicate liquid biopsies.

Among patients treated with ICIs, only one (#17 II) experienced radiological evolution consistent with the definition of hyper-progression (HPD)^15,16^ and died within 12 weeks from the start of ICIs. Its MAFA changed from 0 to 10% from T1 to T2 (Fig. 4). In the overall study population, no other cases of increase in MAFA (T1–T2) superior to 5% and no other cases of hyper-progression were recorded among patients evaluated for longitudinal analysis (Supplementary Table 6). Interestingly, when considering patients with negative plasma *KRAS* at baseline and not experiencing hyper-progression, maximum MAFA increase at T2 was 1%, observed in a patient (#5 III) treated with ICIs, experiencing PD as best RR and died 3 months after the start of treatment (Supplementary Table 6).

On the contrary, even in the presence of high MAFA detected at baseline, we observed RR and persistent clinical benefit in the presence of dramatic reduction of MAFA during treatment (Fig. 4b; Supplementary Table 6).

## Discussion

We report prospective evaluation of liquid biopsy performed at pre-defined time-points with the aim to track biological effects of anti-cancer treatment. Our study represents a proof of concept in the frame of a project aimed to monitor all tumour-specific genetic alterations in plasma and to assess their potential predictive value in patients treated with immunotherapy.

In the present study, the determination and quantification of tumour-specific mutations in plasma are used as surrogate marker of tumour load. We first tested this effect independently of treatment, among all the patients starting a systemic treatment according to clinical practice. We thus found that early evaluation of liquid biopsy is able to anticipate RR and is associated with PFS and OS. Since the treatments performed were heterogeneous, we also tested the hypothesis of a differential effect according to treatment, but the impact of liquid biopsy on outcome overlaps between the two subgroups (chemotherapy *versus* ICIs). This point supports the idea of the dynamic quantification of tumour-specific mutations in plasma to mirror biological behaviour of tumour in a non-invasive manner.

Patients enrolled in the study and carrying *KRAS* mutation in tissue were analysed in plasma using ddPCR, a method characterised by high specificity and sensitivity. The sensitivity found at baseline (48%) is slightly inferior to what has been observed in previous reports.^17,18^ To the best of our knowledge, this is the largest series of lung cancer patients evaluated in both tissue and plasma for the presence of *KRAS* mutation and dynamically analysed during treatment for the presence and quantitative change of *KRAS* mutation. Evidence about *KRAS* detection in plasma is rather limited and potential applications include the detection of acquired resistance mechanisms in *ALK*-rearranged disease.^19,20^ Overall, data about the potential prognostic/predictive role of *KRAS* mutation detected in cfDNA at baseline are controversial.^21,22^ In our experience, the presence of the *KRAS* mutation at baseline has no clear prognostic value in the overall study population, although we cannot exclude that this result might be due to the relatively low number of patients included. It is also not associated with clinical parameters commonly used to identify negative clinical prognostic factors (number of metastatic sites, presence of extra-thoracic disease and presence of liver or bone metastasis).

In our study, dynamic modification of *KRAS* mutation in plasma evaluated at early time-points was associated with outcome endpoints. In particular, patients who are negative at baseline and become positive after 3 or 4 weeks from the start of systemic treatment showed shorter PFS and OS. In this context, the absence of *KRAS* mutation at baseline might be not only related to technical limits but also to biological reasons, such as tumour burden and levels of tumour shedding. In any case, relatively low sensitivity of the method at baseline does not limit the potential applications of the findings, since the increase in MAFA from negative to positive value has the strongest impact on outcome in our experience. The analysis was performed at early time-point and before radiological evaluation and permitted to add information with respect to baseline evaluation. Overall, the results indicate potential usefulness of early monitoring through liquid biopsy and a potential stronger effect of dynamic analysis, when compared to static analysis.

The concept of monitoring tumour burden and therefore biological effects of treatment by analysing circulating biomarkers has been known for long, but only recently the availability of techniques able to detect circulating tumour DNA has opened new perspectives.^5,6^ In lung cancer, the first experiences concern *EGFR*-mutated disease.^17,23–27^ Recently, Kruger et al.^13^ explored the role of *KRAS* mutation in plasma and its changes at different time-points as a marker of early prediction of response to treatment in advanced pancreatic cancer patients treated with first-line chemotherapy. On the other hand, the most useful application of dynamic monitoring of biological effects of treatment in plasma is likely to be in patients treated with ICIs. While results of clinical trials highlight the urgent need to define predictive markers, heterogeneous and dynamic mechanisms can shape immune response during treatment, thus limiting the potential of static predictive markers. A recent report including longitudinal analysis of 15 patients diagnosed with different solid tumours and treated with immunotherapy confirmed the concept that monitoring tumour-specific mutations in plasma may be a useful clinical tool. Patients were monitored at baseline and after 8 weeks, underlying potential applications of early monitoring through plasma genotyping.^11^ In addition, Goldberg et al.^28^ have recently shown that cfDNA response correlates with RR and prolonged survival in a cohort of 28 NSCLC treated with immunotherapy. In parallel, longitudinal evaluation of cfDNA also showed encouraging results in predicting relapse for early-stage disease NSCLCs.^12,29^

In our series, the effect of dynamic variation of liquid biopsy at early time-points is independent of treatment. Indeed, the model was tested for further evaluation among patients treated with different kinds of systemic treatments and confirmed that early evaluation during treatment might affect outcome in both patients treated with chemotherapy and patients treated with ICIs. Anyway, the most important potential applications concern the group of patients treated with ICIs. Among them, baseline evaluation has no prognostic value (Supplementary Fig. 3), but dynamic analysis in plasma at early time-points was able to identify the risk of progression and death. This kind of analysis might provide additional information with respect to radiological imaging, help in the interpretation of RR and anticipating long-term clinical benefit.

The observation that the only patient experiencing hyperprogressive disease had a different dynamic pattern of *KRAS* increase in plasma after 4 weeks also suggests the potential of liquid biopsy in early identification of patients who may have detrimental effect from immunotherapy (Fig. 4).^15,16^ In parallel, patients with extremely elevated levels of MAFA at baseline might drop during ICI treatment and this was associated with a favourable outcome, outlining the importance of longitudinal analyses in the study of further application of liquid biopsy in patients treated with ICIs or ICI plus chemotherapy (Fig. 4).

Measurement of levels of tumour-specific mutations in cfDNA at early time-points could permit to change treatment before radiological assessment and potentially improve outcome, even though further analyses are warranted to confirm the observation in larger case series.

The main limitation of the study is the relatively limited number of patients analysed per type of treatment received. Indeed, larger series treated with homogeneous treatment will permit to draw solid conclusions on the role of this dynamic biomarker. For this reason, we collected samples from a larger cohort of patients treated with ICIs for further analyses, and we are going to test the hypothesis also in patients treated with chemotherapy and pembrolizumab in first-line setting. Another limitation of this study is represented by the fact that we did not assess the influence of other potential predictive biomarkers. In this series, we could not test the impact of co-mutations, such as *STK11/LKB1* mutations, or tumour mutational burden, potentially affecting immunotherapy sensitivity,^30^ due to the low number of samples with available data. Larger homogeneous series of patients treated with immunotherapy will evaluate their impact on our model when detected in both tissue and plasma. Similarly, due to the limited number of samples, we could not integrate the model by including information about PD-L1 expression, and for the same reason we could not analyse the clinical behaviour of different *KRAS* mutations. Follow-up studies will demand exploitation of NGS to track multiple tumour-specific genetic alterations in plasma. We are also retrospectively selecting a group of patients experiencing HPD or early death to confirm our data in this clinical setting. Finally, an interventional clinical trial has been planned to verify whether early assessment of *KRAS* mutation in plasma during treatment can be used to customise first-line systemic treatment and decide between monotherapy and combination therapy with chemotherapy and ICIs. In this context, validation and replacement of the research-use-only ddPCR assay used here with an in vitro diagnostic assay could reduce turnaround time and enable real-world use of this dynamic test.

In conclusion, we demonstrated that tracking *KRAS* mutation in plasma at early time-points during treatment identifies the risk of progression and death in advanced NSCLC. This effect was independent from treatment, but has greater potential applications in patients treated with ICIs. The results open new perspectives in monitoring systemic treatment in advanced non-oncogene-addicted NSCLC.

## Supplementary information

Supplementary Information

## Data Availability

The data generated and analysed during this study are included in this published article and its additional files. Further raw data might be asked to the authors.

## References

[1] Novello S, Barlesi F, Califano R, Cufer T, Ekman S, Levra MG (2016). Metastatic non-small-cell lung cancer: ESMO Clinical Practice Guidelines for diagnosis, treatment and follow-up. Ann. Oncol..

[2] Rolfo C, Mack PC, Scagliotti GV, Baas P, Barlesi F, Bivona TG (2018). Liquid biopsy for advanced non-small cell lung cancer (NSCLC): a statement paper from the IASLC. J. Thorac. Oncol..

[3] Ettinger DS, Wood DE, Aggarwal C, Aisner DL, Akerley W, Bauman JR (2019). NCCN Guidelines Insights: non-small cell lung cancer, version 1.2020. J. Natl Compr. Cancer Netw..

[4] Siravegna G, Marsoni S, Siena S, Bardelli A (2017). Integrating liquid biopsies into the management of cancer. Nat. Rev. Clin. Oncol..

[5] Alix-Panabieres C, Pantel K (2016). Clinical applications of circulating tumor cells and circulating tumor DNA as liquid biopsy. Cancer Discov..

[6] Diehl F, Schmidt K, Choti MA, Romans K, Goodman S, Li M (2008). Circulating mutant DNA to assess tumor dynamics. Nat. Med..

[7] Hu Y, Ulrich BC, Supplee J, Kuang Y, Lizotte PH, Feeney NB (2018). False-positive plasma genotyping due to clonal hematopoiesis. Clin. Cancer Res..

[8] Guibert N, Mazieres J, Delaunay M, Casanova A, Farella M, Keller L (2017). Monitoring of KRAS-mutated ctDNA to discriminate pseudo-progression from true progression during anti-PD-1 treatment of lung adenocarcinoma. Oncotarget.

[9] Yang H, Liang SQ, Schmid RA, Peng RW (2019). New horizons in KRAS-mutant lung cancer: dawn after darkness. Front. Oncol..

[10] Rex, K., Saiki, A. Y., Sun, J.-R., Holt, T., Koppada, N., Lanman, B. A. et al. Abstract 3090: in vivo characterization of AMG 510—a potent and selective KRASG12C covalent small molecule inhibitor in preclinical KRASG12 cancer models. *Cancer Res.*10.1158/1538-7445.AM2019-3090 (2019).

[11] Cabel L, Riva F, Servois V, Livartowski A, Daniel C, Rampanou A (2017). Circulating tumor DNA changes for early monitoring of anti-PD1 immunotherapy: a proof-of-concept study. Ann. Oncol..

[12] Chaudhuri AA, Chabon JJ, Lovejoy AF, Newman AM, Stehr H, Azad TD (2017). Early detection of molecular residual disease in localized lung cancer by circulating tumor DNA profiling. Cancer Discov..

[13] Kruger S, Heinemann V, Ross C, Diehl F, Nagel D, Ormanns S (2018). Repeated mutKRAS ctDNA measurements represent a novel and promising tool for early response prediction and therapy monitoring in advanced pancreatic cancer. Ann. Oncol..

[14] Prentice RL, Williams BJ, Peterson AV (1981). On the regression analysis of multivariate failure time data. Biometrika.

[15] Champiat S, Dercle L, Ammari S, Massard C, Hollebecque A, Postel-Vinay S (2017). Hyperprogressive disease is a new pattern of progression in cancer patients treated by anti-PD-1/PD-L1. Clin. Cancer Res..

[16] Ferrara, R., Mezquita, L., Texier, M., Lahmar, J., Audigier-Valette, C., Tessonnier, L. et al. Hyperprogressive disease in patients with advanced non-small cell lung cancer treated with PD-1/PD-L1 inhibitors or with single-agent chemotherapy. *JAMA Oncol*. 10.1001/jamaoncol.2018.3676 (2018). 10.1001/jamaoncol.2018.3676 PMC6248085 30193240

[17] Sacher AG, Paweletz C, Dahlberg SE, Alden RS, O'Connell A, Feeney N (2016). Prospective validation of rapid plasma genotyping for the detection of EGFR and KRAS mutations in advanced lung cancer. JAMA Oncol..

[18] Reck M, Hagiwara K, Han B, Tjulandin S, Grohe C, Yokoi T (2016). ctDNA determination of EGFR mutation status in European and Japanese patients with advanced NSCLC: the ASSESS Study. J. Thorac. Oncol..

[19] Doebele RC, Pilling AB, Aisner DL, Kutateladze TG, Le AT, Weickhardt AJ (2012). Mechanisms of resistance to crizotinib in patients with ALK gene rearranged non-small cell lung cancer. Clin. Cancer Res..

[20] Bordi P, Tiseo M, Rofi E, Petrini I, Restante G, Danesi R (2017). Detection of ALK and KRAS mutations in circulating tumor DNA of patients with advanced ALK-positive NSCLC with disease progression during crizotinib treatment. Clin. Lung Cancer.

[21] Garzon M, Villatoro S, Teixido C, Mayo C, Martinez A, de Los Llanos Gil M (2016). KRAS mutations in the circulating free DNA (cfDNA) of non-small cell lung cancer (NSCLC) patients. Transl. Lung Cancer Res..

[22] Ai B, Liu H, Huang Y, Peng P (2016). Circulating cell-free DNA as a prognostic and predictive biomarker in non-small cell lung cancer. Oncotarget.

[23] Karachaliou N, Mayo-de las Casas C, Queralt C, de Aguirre I, Melloni B, Cardenal F (2015). Association of EGFR L858R mutation in circulating free DNA with survival in the EURTAC Trial. JAMA Oncol..

[24] Marchetti A, Palma JF, Felicioni L, De Pas TM, Chiari R, Del Grammastro M (2015). Early prediction of response to tyrosine kinase inhibitors by quantification of EGFR mutations in plasma of NSCLC patients. J. Thorac. Oncol..

[25] Yanagita M, Redig AJ, Paweletz CP, Dahlberg SE, O'Connell A, Feeney N (2016). A prospective evaluation of circulating tumor cells and cell-free DNA in EGFR-mutant non-small cell lung cancer patients treated with erlotinib on a phase II trial. Clin. Cancer Res..

[26] Mok T, Wu YL, Lee JS, Yu CJ, Sriuranpong V, Sandoval-Tan J (2015). Detection and dynamic changes of EGFR mutations from circulating tumor DNA as a predictor of survival outcomes in NSCLC patients treated with first-line intercalated erlotinib and chemotherapy. Clin. Cancer Res..

[27] Goto K, Ichinose Y, Ohe Y, Yamamoto N, Negoro S, Nishio K (2012). Epidermal growth factor receptor mutation status in circulating free DNA in serum: from IPASS, a phase III study of gefitinib or carboplatin/paclitaxel in non-small cell lung cancer. J. Thorac. Oncol..

[28] Goldberg SB, Narayan A, Kole AJ, Decker RH, Teysir J, Carriero NJ (2018). Early assessment of lung cancer immunotherapy response via circulating tumor DNA. Clin Cancer Res..

[29] Abbosh C, Birkbak NJ, Wilson GA, Jamal-Hanjani M, Constantin T, Salari R (2017). Phylogenetic ctDNA analysis depicts early-stage lung cancer evolution. Nature.

[30] Skoulidis, F., Goldberg, M. E., Greenawalt, D. M., Hellmann, M. D., Awad, M. M., Gainor, J. F. et al. STK11/LKB1 mutations and PD-1 inhibitor resistance in KRAS-mutant lung adenocarcinoma. *Cancer Discov*. 10.1158/2159-8290.CD-18-0099 (2018). 10.1158/2159-8290.CD-18-0099 PMC6030433 29773717

[31] Bonanno L, Zulato E, Attili I, Pavan A, Del Bianco P, Nardo G (2018). Liquid biopsy as tool to monitor and predict clinical benefit from chemotherapy (CT) and immunotherapy (IT) in advanced non-small cell lung cancer (aNSCLC): a prospective study. Ann. Oncol..

