# Supplementary material for: Early assessment of KRAS mutation in cfDNA correlates with risk of progression and death in advanced non-small-cell lung cancer
Source: Br J Cancer. 2020 May 7;123(1):81–91. doi: 10.1038/s41416-020-0833-7 (PMC7341732; doi:10.1038/s41416-020-0833-7)
Supplement: Supplementary file 1 — Supplementary Information [file 41416_2020_833_MOESM1_ESM.pdf]

SUPPLEMENTARY INFORMATION

SUPPLEMENTARY FIGURE LEGENDS

Supplementary Figure 1. Study design.

Time point of plasma samples collection and cfDNA analysis (A). Flow chart of study population (B).

Supplementary Figure 2. Association of presence of *KRAS* mutation at T2 and radiological response. Radiological response assessed by RECIST criteria v1.1. (PD: Progressive Disease; SD: stable disease; PR: Partial Response) according to the presence or the absence of *KRAS* mutation at T2. Black and white diamonds indicate systemic treatment with CT or ICIs, respectively.

Supplementary Figure 3. Post-hoc analyses. Post-hoc analyses of radiological response, progression-free survival and overall survival for the presence of *KRAS* mutation and the variation in mutated allele fractional abundance (MAFA) of *KRAS* mutation in CT or ICIs subgroups.

Supplementary Figure 4. Association of liquid biopsy results and outcome in patients receiving Chemotherapy (CT).

Progression-free survival (PFS) and overall survival (OS) in patients receiving Chemotherapy (CT). PFS according to the presence of *KRAS* mutation at T2 (A); PFS according to the presence of *KRAS* mutation at T3 (B); PFS according to MAFA variation from T1 to T2 (C); PFS according to MAFA variation from T1 to T3 (D); OS according to the presence of *KRAS* mutation at T2 (E); OS according to the presence of *KRAS* mutation at T3 (F); OS according to MAFA variation from T1 to T2 (G); OS according to MAFA variation from T1 to T3 (H). The hazard ratios with 95% confidence interval and p-values are also reported in figure.

Supplementary Figure 5. Association of liquid biopsy results and outcome in patients receiving immune checkpoint inhibitors (ICIs).

Progression-free survival (PFS) and overall survival (OS) in patients receiving immune checkpoint inhibitors (ICIs).

PFS according to the presence of *KRAS* mutation at T2 (A); PFS according to the presence of KRAS mutation at T3 (B); PFS according to MAFA variation from T1 to T2 (C); PFS according to MAFA variation from T1 to T3 (D); OS according to the presence of *KRAS* mutation at T2 (E); OS according to the presence of *KRAS* mutation at T3 (F); OS according to MAFA variation from T1 to T2 (G); OS according to MAFA variation from T1 to T3 (H). The hazard ratios with 95% confidence interval and p-values are also reported in figure.

Supplementary Fig. 1

A

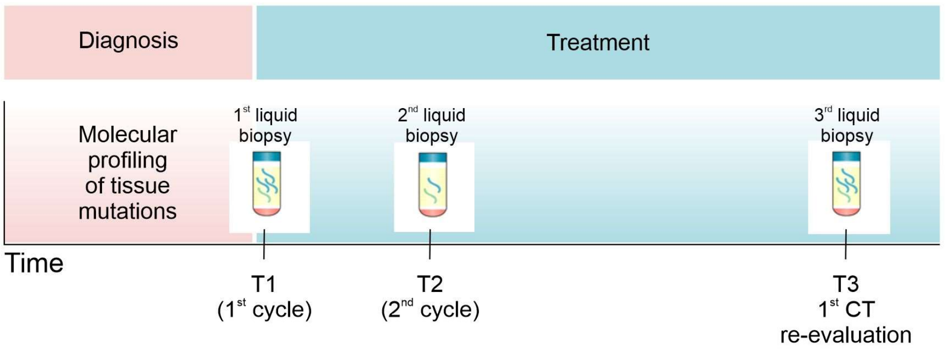

B

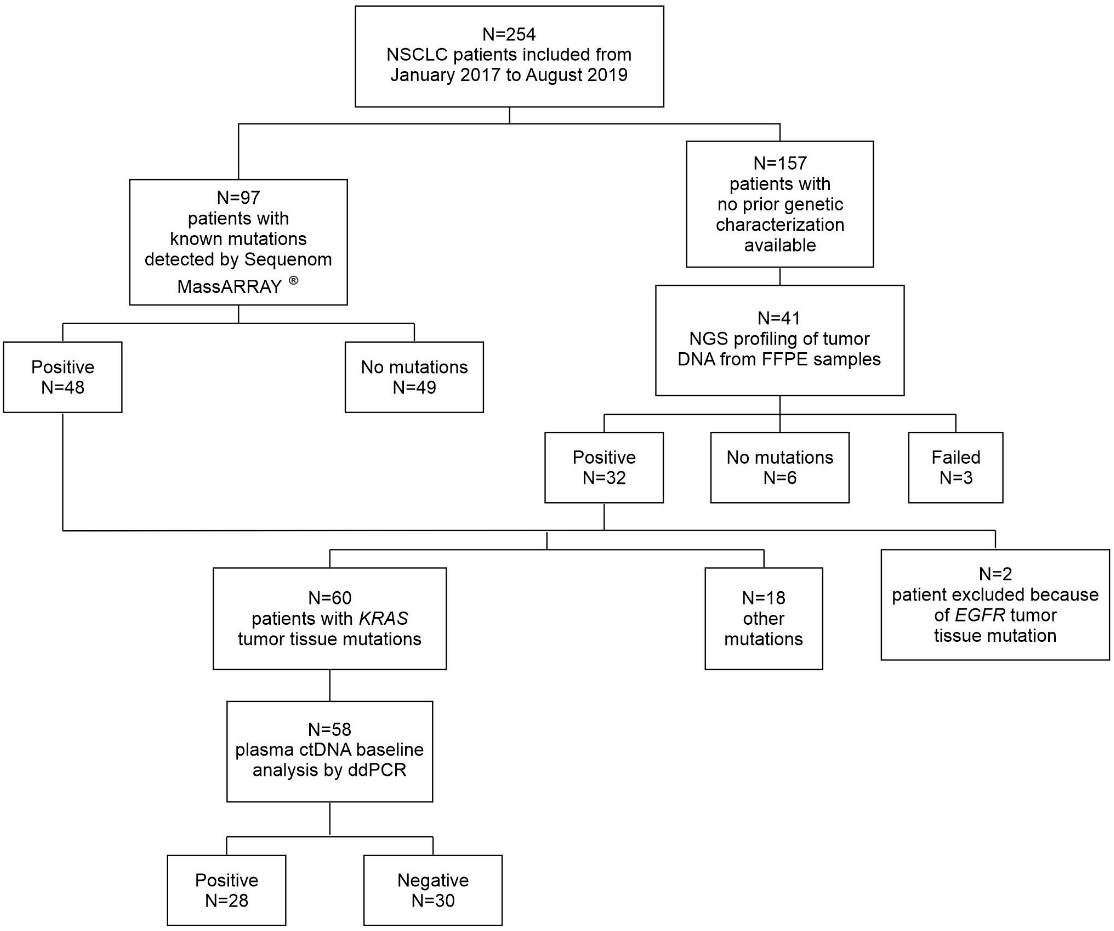

Supplementary Fig. 2

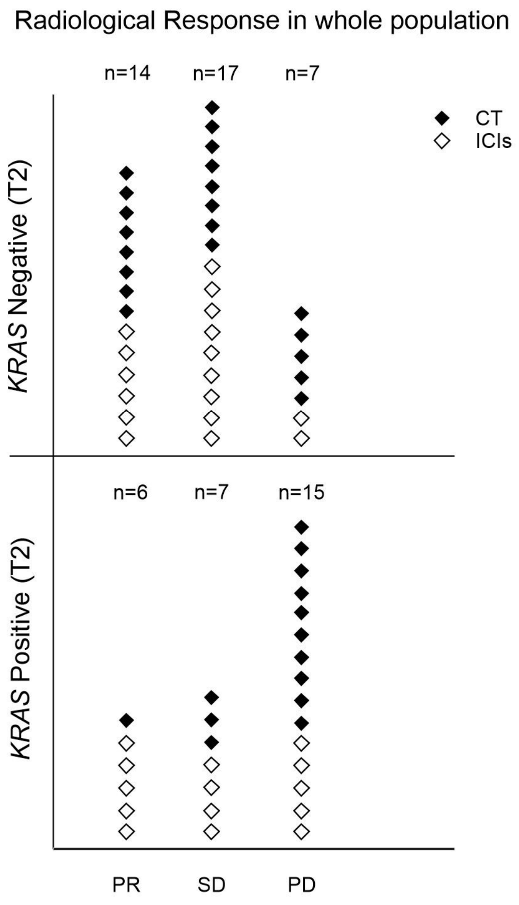

Supplementary Fig. 3

	KRAS positive MAFA increase Events/N	(%)	*KRAS* negative MAFA stable/ decrease Events/N	(%)		*p*-value for interaction*
**RADIOLOGICAL RESPONSE**					OR (95% CI)	
**Mutation at T1**						0.2954
chemotherapy	12/19	(63.2)	7/20	(35.0)	3.17 (0.99, 10.2)	
immune checkpoint inhibitors	4/16	(25.0)	4/17	(23.5)	1.10 (0.22, 5.40)	
**Mutation at T2**						0.6837
chemotherapy	10/14	(71.4)	5/21	(23.8)	7.70 (2.06, 28.8)	
immune checkpoint inhibitors	5/14	(35.7)	2/17	(11.8)	4.66 (0.71, 30.7)	
**Mutation at T3**						0.1309
chemotherapy	8/12	(66.7)	4/20	(20.0)	8.00 (1.57, 40.6)	
immune checkpoint inhibitors	4/9	(44.4)	0/20	(0.0)	. (. , .)	
**Variation (T1-T2)**						0.9435
chemotherapy	4/5	(80.0)	11/30	(36.7)	8.58 (0.62, 119)	
immune checkpoint inhibitors	5/12	(41.7)	2/19	(10.5)	7.75 (1.03, 58.2)	
**Variation (T1-T3)**						0.8806
chemotherapy	7/9	(77.8)	5/23	(21.7)	12.6 (2.02, 78.6)	
immune checkpoint inhibitors	3/7	(42.9)	1/22	(4.5)	15.8 (1.29, 193)	
**PROGRESSION FREE SURVIVAL**					HR (95% CI)	
**Mutation at T1**						0.2544
chemotherapy	18/19	(94.7)	18/20	(90.0)	1.39 (0.69, 2.78)	
immune checkpoint inhibitors	10/16	(62.5)	15/18	(83.3)	0.76 (0.35, 1.67)	
**Mutation at T2**						0.9942
chemotherapy	13/14	(92.9)	19/21	(90.5)	1.52 (0.63, 3.66)	
immune checkpoint inhibitors	11/14	(78.6)	12/18	(66.7)	1.52 (0.68, 3.37)	
**Mutation at T3**						0.0794
chemotherapy	11/12	(91.7)	18/20	(90.0)	1.76 (0.61, 5.04)	
immune checkpoint inhibitors	9/9	(100)	12/20	(60.0)	5.46 (2.49, 11.9)	
**Variation (T1-T2)**						0.9265
chemotherapy	5/5	(100)	27/30	(90.0)	2.57 (0.93, 7.12)	
immune checkpoint inhibitors	11/12	(91.7)	12/20	(60.0)	2.41 (1.08, 5.38)	
**Variation (T1-T3)**						0.2062
chemotherapy	8/9	(88.9)	21/23	(91.3)	2.14 (0.61, 7.60)	
immune checkpoint inhibitors	7/7	(100)	14/22	(63.6)	5.40 (2.48, 11.7)	
**OVERALL SURVIVAL**					HR (95% CI)	
**Mutation at T1**						0.0892
chemotherapy	14/19	(73.7)	5/20	(25.0)	4.23 (1.49, 12.0)	
immune checkpoint inhibitors	5/16	(31.3)	6/18	(33.3)	1.03 (0.31, 3.41)	
**Mutation at T2**						0.3418
chemotherapy	10/14	(71.4)	5/21	(23.8)	4.55 (1.48, 13.9)	
immune checkpoint inhibitors	6/14	(42.9)	4/18	(22.2)	2.00 (0.60, 6.73)	
**Mutation at T3**						0.1750
chemotherapy	7/12	(58.3)	5/20	(25.0)	3.66 (0.91, 14.8)	
immune checkpoint inhibitors	6/9	(66.7)	1/20	(5.0)	20.3 (2.77, 149)	
**Variation (T1-T2)**						0.2304
chemotherapy	4/5	(80.0)	11/30	(36.7)	8.28 (2.12, 32.3)	
immune checkpoint inhibitors	6/12	(50.0)	4/20	(20.0)	2.61 (0.80, 8.51)	
**Variation (T1-T3)**						0.5982
chemotherapy	6/9	(66.7)	6/23	(26.1)	3.49 (0.89, 13.7)	
immune checkpoint inhibitors	4/7	(57.1)	3/22	(13.6)	5.83 (1.50, 22.6)	

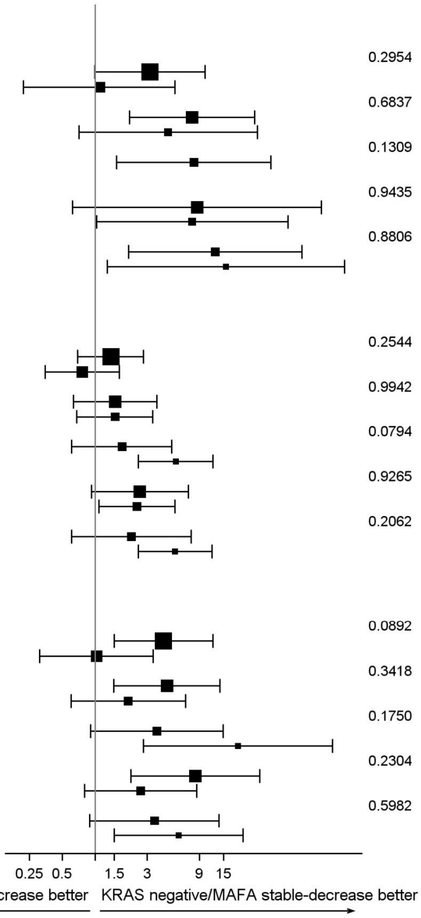

* Unadjusted for multiplicity

Supplementary Fig.4

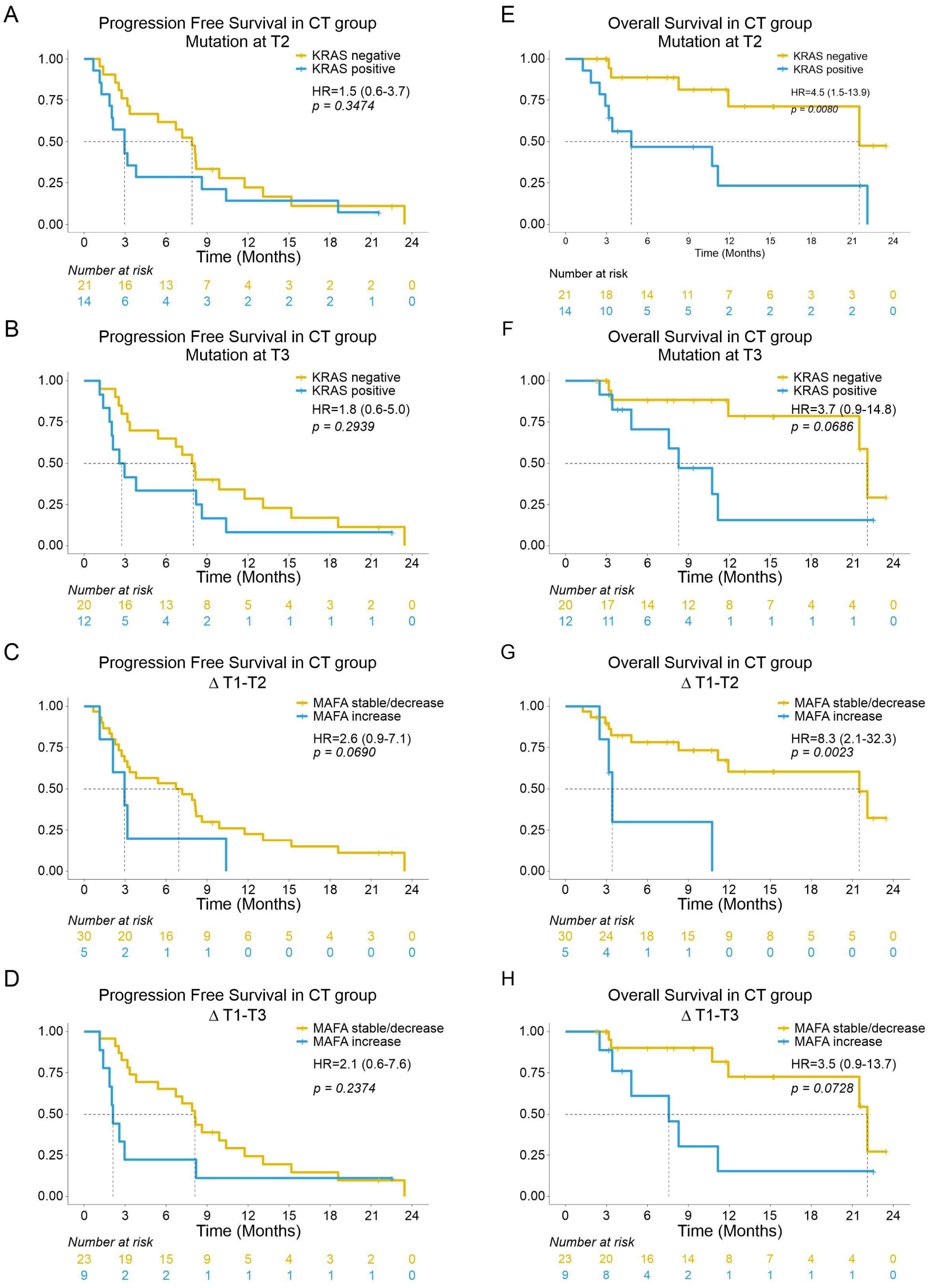

Supplementary Fig. 5

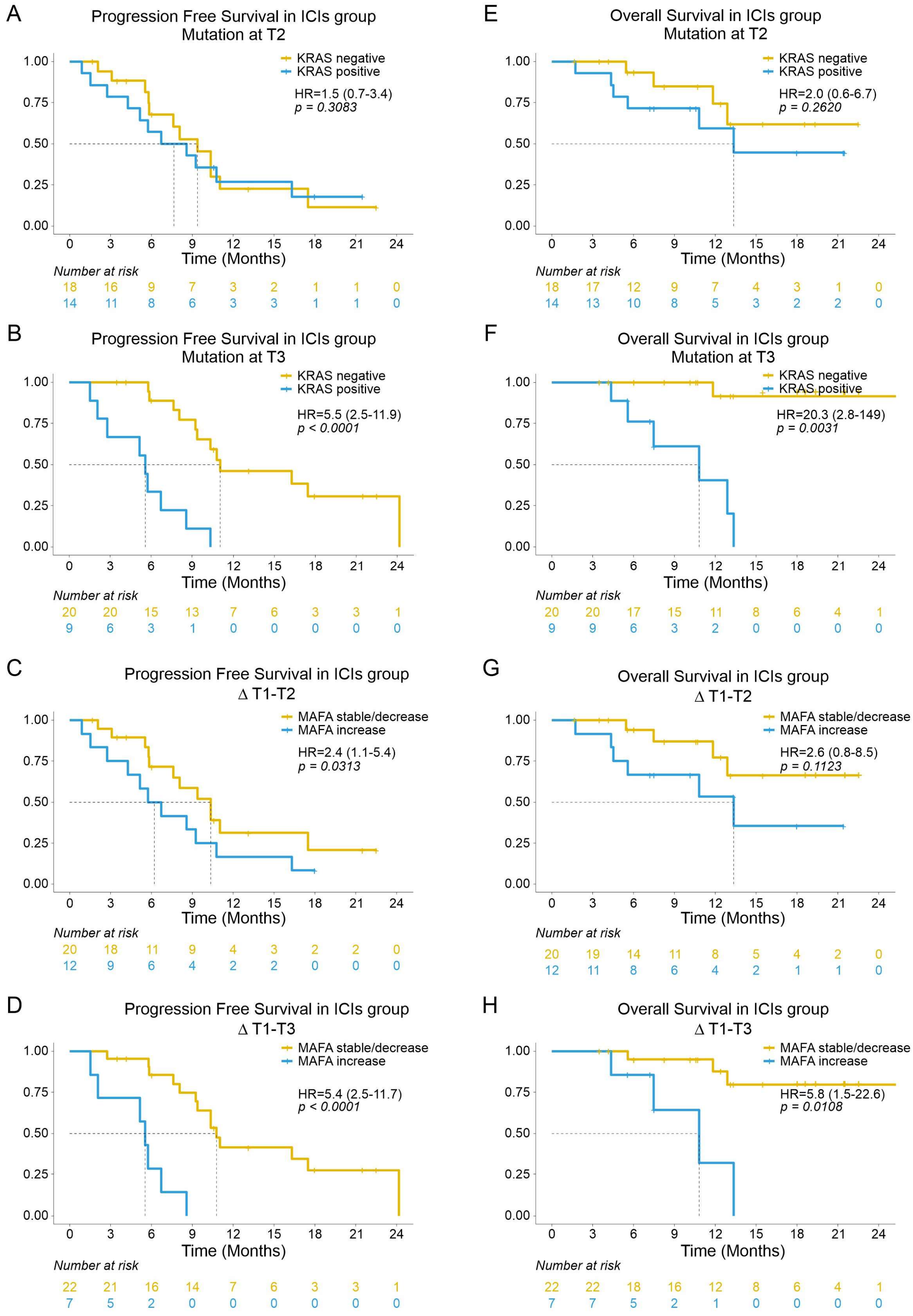

SUPPLEMENTARY TABLES

Supplementary Table 1. Genes included in Sequenom MassARRAY® (A) and NGS (B) panels for tumor tissue genotyping.

A.

**Sequenom MassARRAY® panel (Myriapod® Lung status kit)**		
10 genes (307 nucleotide variants)		
EGFR	*PIK3CA*	*ERBB2*
KRAS	*NRAS*	*DDR2*
BRAF	*ALK*	*MAP2K1*
RET		

B.

**Targeted NGS panel**		
30 genes		
AKT1	*FGFR2*	*NTRK1*
ALK	*FGFR3*	*NTRK2*
BRAF	*FGFR4*	*NTRK3*
DDR2	*JAK2*	*PIK3CA*
EGFR	*KRAS*	*PTCH1*
EPHA3	*MAP2K1*	*PTEN*
EPHA5	*MET*	*PTPN11*
ERBB2	*NOTCH1*	*PTPRD*
FBXW7	*NRAS*	*STK11*
FGFR1	*NRF2*	*TP53*

Supplementary Table 2. Mutations detected in tumor samples in the whole study population.

Patient	Mutation detected in tumor tissue	Detection technique
1	*BRAF* p.V600E	Sequenom MA
3	*KRAS* p.G12D	Sequenom MA
4	*KRAS* p.G12A	NGS
5	*KRAS* p.Q61R	Sequenom MA
8	Failed	NGS
9	*KRAS* p.G12F	NGS
10	*KRAS* p.G12C	Sequenom MA
13	Failed	NGS
14	*KRAS* p.G13D	Sequenom MA
15	*KRAS* p.G13D	Sequenom MA
17	*KRAS* p.G12D	Sequenom MA
19	*EGFR* p.D770-N771insSVD	Sequenom MA
20	*BRAF* p.G466V	NGS
21	*TP53* p.C242F	NGS
22	No mutation detected	Sequenom MA
24	No mutation detected	NGS
26	*KRAS* p.G12	NGS
27	No mutation detected	Sequenom MA
28	*KRAS* p.G12C	Sequenom MA
29	Failed	NGS
30	*KRAS* p.G12D; TP53 p.R158L	NGS
32	*ERBB2* p.P780_Y781insGSP	NGS
33	No mutation detected	Sequenom MA
35	*KRAS* p.Q61L	Sequenom MA
37	*EGFR* p.D770-N771insSVD	Sequenom MA
38	No mutation detected	NGS
40	*KRAS* p.Q61H	Sequenom MA
41	*KRAS* p.G12D	Sequenom MA
42	*KRAS* p.G12C	Sequenom MA
45	*KRAS* p.G12C	Sequenom MA
48	*KRAS* p.G12A	Sequenom MA
49	*TP53* p.R175H	NGS
50	*KRAS p.G12S* ; TP53 p.G266E	NGS
51	No mutation detected	Sequenom MA
54	No mutation detected	Sequenom MA
55	No mutation detected	NGS
56	*BRAF* p.D594N	NGS
57	*EGFR* p.D770-N771insSVD	Sequenom MA
61	*EGFR* p.E709K	NGS
64	*MET* p.D1028H	NGS

66	*KRAS* p.G12C	Sequenom MA
68	*KRAS* p.G12V	NGS
69	*ERBB2* p.A775V	NGS
70	*KRAS* p.G12C	Sequenom MA
73	*KRAS* p.G12D	Sequenom MA
75	*KRAS* p.G12C	Sequenom MA
76	*KRAS* p.G12D	NGS
78	No mutation detected	Sequenom MA
80	No mutation detected	Sequenom MA
81	No mutation detected	Sequenom MA
82	No mutation detected	Sequenom MA
83	No mutation detected	Sequenom MA
84	No mutation detected	Sequenom MA
85	*KRAS* p.G12C	NGS
86	*KRAS* p.G12C	Sequenom MA
87	No mutation detected	Sequenom MA
90	*KRAS* p.G13D	NGS
91	*EGFR* p.P848L	NGS
94	No mutation detected	Sequenom MA
98	No mutation detected	Sequenom MA
103	*KRAS* p.G12C	Sequenom MA
104	*KRAS* p.G13D	Sequenom MA
106	*EGFR* p.L858R	NGS
107	*KRAS* p.G13S	NGS
112	*KRAS* p.G12A	Sequenom MA
113	No mutation detected	Sequenom MA
114	*KRAS* p.Q61H	Sequenom MA
117	No mutation detected	Sequenom MA
119	No mutation detected	Sequenom MA
120	No mutation detected	Sequenom MA
122	*KRAS* p.Q61H	Sequenom MA
123	No mutation detected	Sequenom MA
124	*PIK3CA* p.E545K	Sequenom MA
126	*KRAS* p.G12A	Sequenom MA
128	No mutation detected	Sequenom MA
130	*ERBB2* Amplification	NGS
132	No mutation detected	Sequenom MA
136	*MET* p.D1028H	NGS
139	*KRAS* p.G12V	Sequenom MA
140	*KRAS* p.G12C	Sequenom MA
141	No mutation detected	Sequenom MA
146	*KRAS* p.G12A	Sequenom MA
152	*KRAS* p.Q61H	Sequenom MA
154	*KRAS* p.G12S	Sequenom MA

155	*KRAS* p.G12C	NGS
157	No mutation detected	Sequenom MA
159	No mutation detected	Sequenom MA
162	No mutation detected	Sequenom MA
163	*BRAF* p.N581S	NGS
174	*KRAS* p.G12C	Sequenom MA
178	*KRAS* p.G12C	NGS
182	No mutation detected	Sequenom MA
187	*KRAS* p.Q61H	Sequenom MA
190	*EGFR* p.L858R	Sequenom MA
196	*KRAS* p.G12C	Sequenom MA
198	No mutation detected	Sequenom MA
203	No mutation detected	Sequenom MA
205	No mutation detected	Sequenom MA
206	*KRAS* p.G12D	Sequenom MA
209	*KRAS* p.G12C	Sequenom MA
210	*KRAS* p.G12V	Sequenom MA
211	*KRAS* p.G12C	Sequenom MA
212	No mutation detected	Sequenom MA
215	*BRAF* p.V600E	Sequenom MA
216	No mutation detected	Sequenom MA
217	No mutation detected	NGS
218	No mutation detected	Sequenom MA
219	No mutation detected	Sequenom MA
222	*KRAS* p.G12D	Sequenom MA
223	No mutation detected	Sequenom MA
226	No mutation detected	Sequenom MA
229	*KRAS* p.G12A	NGS
230	No mutation detected	Sequenom MA
231	No mutation detected	Sequenom MA
232	*KRAS* p.G12A	NGS
233	*KRAS* p.G12C	Sequenom MA
236	No mutation detected	NGS
238	No mutation detected	Sequenom MA
240	No mutation detected	Sequenom MA
244	*KRAS* p.G12V	Sequenom MA
248	*KRAS* p.G12D	Sequenom MA
250	*KRAS* p.G12A	NGS
251	*KRAS* p.G12A	NGS
257	No mutation detected	Sequenom MA
258	*KRAS* p.G12C	Sequenom MA
260	No mutation detected	Sequenom MA
265	*KRAS* p.G12V	Sequenom MA
266	No mutation detected	Sequenom MA

268	No mutation detected	Sequenom MA
270	No mutation detected	NGS
273	No mutation detected	Sequenom MA
274	*KRAS* p.G12D	NGS
278	No mutation detected	Sequenom MA
285	No mutation detected	Sequenom MA
290	No mutation detected	Sequenom MA
291	*KRAS* p.G12R	NGS
301	No mutation detected	Sequenom MA
317	No mutation detected	Sequenom MA

138 tumor tissue samples were genetically screened. 60 patients resulted *KRAS* mutated.

Sequenom MA: Sequenom MassARRAY ®; NGS: next-generation sequencing.

Supplementary Table 3. Clinical factors affecting radiological response (RR) in the study population.

		PD/N	OR (PD)	95%CI	*p-value*
**Age (years)**	Continuous	27/72	1.0	0.9-1.0	*0.0894*
**Gender**	Male	12/37	0.6	0.2-1.7	*0.3665*
	Female	15/35	1		
**Smoking**	No	5/8	1		
	Ex	9/36	0.2	0.0-1.0	*0.0459*
	Yes	13/28	0.5	0.1-2.7	*0.4500*
**PS**	0	13/36	1		
	1-2	14/36	1.1	0.4-3.0	*0.8004*
**Extrathoracic site**	No	8/30	1		
	1	10/24	2.0	0.7-5.6	*0.2048*
	>1	9/18	2.8	0.8-9.2	*0.1020*
**number of metastatic sites**	0-1	8/37	1		
	2-4	19/35	4.3	1.5-11.8	***0.0053***
**Bone/Liver**	No	14/46	1		
	Bone	5/9	2.9	0.7-12.3	*0.1575*
	Liver	5/9	2.9	0.7-12.5	*0.1590*
	Both	3/8	1.4	0.3-6.8	*0.7143*
**Therapy**	CT	19/39	2.9	1.1-7.6	***0.0253***
	IT	8/33	1		
**Line**	1	13/43	1		
	>1	14/29	2.2	0.8-5.8	*0.1244*
**PD-L1**	0	9/19	1		
	1-49	3/15	0.8	0.5-1.1	*0.1957*
	>50	1/16	0.1	0.0-0.9	*0.0431*

Supplementary Table 4. Clinical factors affecting Progression Free Survival (PFS) in the study population.

		PD/N	Median PFS (95%CI)	HR	95%CI	*p-value*
	**Overall**	61/73	**6.7 (4.3-8.6)**			
**Line of Treatment**	First	48/58				
	Second	10/12				
	Third	3/3				
**Age (years)**	Continuous			1.0	0.9-1.0	*0.3489*
**Gender**	Male	31/37	8.2 (4.3-10.4)	0.8	0.5-1.3	*0.3769*
	Female	30/36	5.4 (3.0-7.9)	1		
**Smoking**	Yes	24/29	5.8 (2.1-8.2)	1.2	0.4-3.5	*0.7950*
	Ex	31/36	7.9 (5.4-10.4)	0.8	0.3-2.5	*0.7560*
	No	6/8	3.0 (2.0-)	1		
**PS**	0	30/37	7.9 (3.2-10.3)	1		
	1-2	31/36	5.8 (3.1-8.6)	0.9	0.6-1.5	*0.7084*
**Extrathoracic site**	No	23/31	8.1 (5.8-11.7)	1		
	1	21/24	6.2 (2.8-10.3)	1.4	0.8-2.5	*0.2526*
	>1	17/18	3.6 (2.0-7.6)	1.8	0.9-3.7	*0.0909*
**number of metastatic sites**	0-1	29/38	8.2 (6.7-11.7)	1		
	2-4	32/35	3.4 (2.8-5.6)	2.0	1.1-3.4	***0.0164***
**Bone/Liver**	No	36/47	8.2 (5.8-10.8)	1		
	Bone	9/9	3.0 (0.2-16.3)	2.3	1.0-5.4	*0.0468*
	Liver	8/9	5.2 (2.5-10.3)	1.9	0.9-4.0	*0.0851*
	Both	8/8	4.7 (0.9-8.6)	2.1	0.9-4.9	*0.0684*
**Therapy**	CT	36/39	3.4 (2.5-8.1)	1.9	1.2-3.2	***0.0084***
	IT	25/34	8.6 (5.7-10.8)	1		
**Line**	1	35/43	8.1 (3.8-10.3)	1		
	>1	26/30	5.6 (2.6-7.6)	1.2	0.6-2.5	*0.5626*
**PD-L1**	0	18/20	6.7 (2.5-8.1)	1		
	1-49	12/15	7.6 (3.2-13.1)	0.4	0.1-0.8	***0.0192***
	>50	9/16	10.8 (8.2-)	0.3	0.1-0.5	***0.0002***

Supplementary Table 5. Clinical factors affecting Overall Survival (OS) in the study population.

		Death/N	Median OS (95%CI)	HR	95%CI	*p-value*
	**Overall**	**30/73**	**13.3 (10.8-)**			
	**First**	**23/58**				
	**Second**	**5/12**				
	**Third**	**2/3**				
**Age (years)**	Continuous			1.0	0.9-1.0	*0.2966*
**Gender**	Male	15/37	21.5 10.7-)	0.9	0.4-1.9	*0.8186*
	Female	15/36	11.8 (7.5-)	1		
**Smoking**	Yes	10/29	13.3 (8.3-)	0.9	0.4-1.8	*0.7403*
	No-Ex	20/44	12.9 (10.7-)	1		
**PS**	0	12/37	-	1		
	1-2	18/36	13.3 (7.6-)	1.3	0.6-2.7	*0.4698*
**Extrathoracic**	No	11/31	21.5 (11.2-)	1		
**site**	1	10/24	-	1.2	0.5-2.8	*0.6521*
	>1	9/18	10.7 (3.4-)	2.1	0.9-4.8	*0.0899*
**number of metastatic sites**	0-1	12/38	22.1 (11.8-)	1		
	2-4	18/35	7.6 (4.8-13.3)	2.5	1.1-5.7	***0.0227***
**Bone/Liver**	No	19/47	21.5 (11.2-)	1		
	Bone	5/9	6.5 (0.6-)	2.4	0.8-7.2	*0.1248*
	Liver	2/9	-	0.5	0.1-1.8	*0.2581*
	Both	4/8	10.7 (1.7-13.3)	1.9	0.8-4.6	*0.1500*
**Therapy**	CT	19/39	11.9 (6.5-22.1)	2.0	1.0-4.1	***0.0563***
	IT	11/34	-	1		
**Line**	1	18/43	21.5 (11.8-)	1		
	>1	12/30	10.7 (5.4-)	1.0	0.4-2.7	*0.9747*
**PD-L1**	0	7/20	11.9 (4.8-)	1		
	1-49	4/15	-	0.6	0.2-2.3	*0.4704*
	>50	5/16	-	0.6	0.2-1.7	*0.3117*

Supplementary Table 6. Semi-quantitative evaluation of *KRAS* mutations in plasma at different time-points and radiological response.

Patient	Mutation in tissue	1st liquid biopsy (MAFA)	2nd liquid biopsy (MAFA)	3rd liquid biopsy (MAFA)	Therapy	Radiological Response
#3	*KRAS* G12D	6,40%	0,00%	0,00%	CT	SD
#4	*KRAS* G12A	0,48%	0,30%	15,00%	CT	PD
#5	*KRAS* Q61R	0,00%	0,00%	0,00%	CT	PR
#5 II	*KRAS* Q61R	0,00%	0,00%	0,00%	CT	PD
#5 III	*KRAS* Q61R	0,00%	1,00%	2,00%	ICIs	SD
#10	*KRAS* G12C	1,25%	1,06%		CT	PD
#14	*KRAS* G13D	0,70%	0,00%	59,60%	CT	SD
#15	*KRAS* G13D	0,00%	0,00%	0,00%	CT	SD
#17	*KRAS* G12D	0,00%	0,00%	0,00%	CT	PD
**#17 II**	***KRAS* G12D**	**0,00%**	**10,80%**		ICIs	PD
#26	*KRAS* G12C	0,00%	1,80%		CT	PD
#28	*KRAS* G12C	0,87%	0,21%	0,00%	CT	SD
#30	*KRAS* G12D	0,00%	0,60%	0,00%	ICIs	PR
#30 II	*KRAS* G12D	28,00%			CT	PD
#35	*KRAS* Q61L	8,10%	0,95%	2,60%	CT	PD
#35 II	*KRAS* Q61L	0,60%	0,00%	1,40%	ICIs	SD
#35 III	*KRAS* Q61L	17,50%	21,90%	0,80%	CT	SD
#40	*KRAS* Q61R	0,00%	0,00%	0,57%	ICIs	PD
#41	*KRAS* G12D	0,00%	n.e.	0,00%	ICIs	RP
#42	*KRAS* G12C	0,00%	0,00%	0,00%	CT	PR
#42 II	*KRAS* G12C	0,00%	0,00%	0,00%	CT	PD
#42 III	*KRAS* G12C	0,00%	0,00%	0,00%	ICIs	SD
#45	*KRAS* G12C	0,00%	0,00%	1,30%	CT	SD
#48	*KRAS* G12A	4,00%	0,80%	4,80%	CT	PD
#50	*KRAS* G12S	1,00%	1,60%	3,50%	ICIs	PD
#50 II	*KRAS* G12S	11,20%	n.e.	32,20%	CT	PD
#66	*KRAS* G12C	0,00%			CT	PD
#68	*KRAS* G12V	0,00%	0,00%	0,00%	CT	PR
#68 II	*KRAS* G12V	0,00%	0,00%	0,00%	ICIs	SD
#70	*KRAS* G12C	10,30%	4,10%	9,00%	CT	SD
#70 II	*KRAS* G12C	4,30%	5,80%		ICIs	PD
#73	*KRAS* G12D	13,30%	3,80%		CT	PD
#75	*KRAS* G12C	1,00%	1,50%	6,20%	CT	PD
#76	*KRAS* G12D	0,80%	5,30%	3,50%	CT	PD
#85	*KRAS* G12C	14,20%	6,00%	0,00%	CT	PR
#86	*KRAS* G12C	0,00%	0,00%	0,00%	CT	PR
#90	*KRAS* G13D	33,50%	32,00%		CT	PD

#103	*KRAS*	G12C	1,80%	2,90%	5,20%	ICIs	SD
#104	*KRAS*	G13D	0,00%	0,00%	0,00%	ICIs	PR
#112	*KRAS*	G12A	32,80%	1,20%	0,00%	ICIs	PR
#114	*KRAS*	G12A	3,90%	9,50%	0,00%	ICIs	PR
#122	*KRAS*	Q61H	0,00%	0,00%	0,00%	CT	PR
#126	*KRAS*	G12A	0,00%	0,00%	1,80%	CT	PD
#126 II	*KRAS*	G12A	0,59%	0,90%	0,92%	CT	PD
#139	*KRAS*	G12V	0,00%	0,00%	0,00%	CT	SD
#140	*KRAS*	G12C	26,70%			CT	PD
#146	*KRAS*	G12A	11,70%	0,00%	0,00%	ICIs	PR
#152	*KRAS*	Q61H	0,00%	0,00%	0,00%	CT	RP
#152 II	*KRAS*	Q61H	0,00%	0,00%	0,00%	ICIs	SD
#154	*KRAS*	G12S	0,00%	0,00%		ICIs	PD
#155	*KRAS*	G12C	0,00%	0,00%	0,00%	CT	SD
#174	*KRAS*	G12C	0,52%	1,90%	0,00%	ICIs	PR
#178	*KRAS*	G12C	0,81%	0,00%	0,80%	ICIs	PR
#187	*KRAS*	Q61H	0,00%	0,00%	0,00%	CT	SD
#187 II	*KRAS*	Q61H	0,00%	0,00%		ICIs	n.e.
#196	*KRAS*	G12C	0,60%	0,00%	0,00%	CT	PR
#196 II	*KRAS*	G12C	2,10%	0,00%	0,00%	ICIs	PR
#206	*KRAS*	G12D	0,00%	0,00%	0,00%	ICIs	SD
#209	*KRAS*	G12C	0,00%			ICIs	PD
#210	*KRAS*	G12V	0,00%	0,00%	0,00%	CT	PD
#211	*KRAS*	G12C	0,00%	0,00%	0,00%	CT	SD
#222	*KRAS*	G12D	0,46%	0,70%	0,00%	ICIs	SD
#229	*KRAS*	G12A	0,00%	0,00%	0,00%	CT	PR
#232	*KRAS*	G12A	3,00%	3,90%	3,60%	ICIs	PD
#233	*KRAS*	G12C	0,00%	0,00%	0,00%	ICIs	PR
#244	*KRAS*	G12V	0,00%	0,00%	0,00%	ICIs	PR
#248	*KRAS*	G12D	12,30%	14,50%	8,20%	ICIs	PD
#250	*KRAS*	G12A	0,00%	0,00%	0,00%	ICIs	SD
#251	*KRAS*	G12A	53,70%	10,80%	0,00%	ICIs	PR
#258	*KRAS*	G12C	0,00%	0,00%	0,00%	ICIs	SD
#265	*KRAS*	G12V	0,00%	3,20%	1,10%	ICIs	SD
#274	*KRAS*	G12D	1,00%	0,00%	0,00%	ICIs	SD
#291	*KRAS*	G12R	1,20%	0,00%	0,00%	ICIs	SD

Comparison of molecular alterations found in tumor tissue and in baseline cfDNA (n=58 *KRAS* mutated patients), and changes in fractional abundance of mutation in plasma samples at different time points. When patients enrolled in the study after PD started a new systemic treatment, they were re-considered for longitudinal analysis (n=73). Romanic numbers (II or III) indicate the start of a new treatment during the period of observation. The only case receiving ICIs and experiencing hyper-progression is highlighted in bold (#17 II).

MAFA: mutated allele fractional abundance; CT: chemotherapy; ICIs: immune checkpoint inhibitors; n.e.: not evaluable; PD: progressive Disease; SD: stable disease; PR: partial response

Supplementary Table 7. Association between mutation at baseline (T1) and disease burden.

		No mutation	Mutation	Total	OR mutation (95%CI)	*p-value*
**Extrathoracic**	No	19 (50.0%)	12 (34.3%)	31 (42.5%)	1	
**site**	1	13 (34.2%)	11 (31.4%)	24 (32.9%)	2.6 (0.6-11.2)	*0.2004*
	>1	6 (15.8%)	12 (34.3%)	18 (24.7%)	3.4 (0.9-12.5)	*0.0698*
**number of metastatic sites**	0-1	23 (60.5%)	15 (42.9%)	38 (52.0%)	1	
	2-4	15 (39.5%)	20 (57.1%)	35 (48.0%)	1.9 (0.8-4.5)	*0.1521*
**Bone/Liver**	No	26 (68.4%)	21 (60.0%)	47 (64.4%)	1	
	Bone	2 (5.3%)	7 (20.0%)	9 (12.3%)	3.9 (0.7-21.1)	*0.1145*
	Liver	6 (15.8%)	3 (8.6%)	9 (12.3%)	0.6 (0.1-3.4)	*0.5278*
	Both	4 (10.5%)	4 (11.4%)	8 (11.0%)	0.7 (0.1-5.0)	*0.7635*
**Line number**	1	21 (55.3%)	22 (62.9%)	43 (58.9%)	1	
	>1	17 (44.7%)	13 (37.1%)	30 (41.1%)	1.2 (0.6-2.2)	*0.6131*
**Total**		**38**	**35**	**73**		
